# Retraction Note: Brown adipose tissue-derived Nrg4 alleviates endothelial inflammation and atherosclerosis in male mice

**DOI:** 10.1038/s42255-025-01344-4

**Published:** 2025-07-02

**Authors:** Lingfeng Shi, Yixiang Li, Xiaoli Xu, Yangyang Cheng, Biying Meng, Jinling Xu, Lin Xiang, Jiajia Zhang, Kaiyue He, Jiayue Tong, Junxia Zhang, Lingwei Xiang, Guangda Xiang

**Affiliations:** 1 Department of Endocrinology, General Hospital of Central Theater Command, Wuhan, China; 2 The First School of Clinical Medicine, Southern Medical University, Guangzhou, China; 3 Endocrinology Department, The First Affiliated Hospital of the Army Medical University (Third Military Medical University), Chongqing, China; 4 Department of Hematology and Medical Oncology, School of Medicine, Emory University, Atlanta, GA USA; 5 Centers for Surgery and Public Health, Brigham and Women’s Hospital, Boston, MA USA

Retraction to: *Nature Metabolism* 10.1038/s42255-022-00671-0, published online 18 November 2022.

The Editors have retracted this article. After publication, concerns were raised regarding some of the data presented in the Figures and Extended Data Figures, specifically:

- Fig. 1e KO-WD image appears to overlap with Extended Data Fig. 4D BATT(KO);
- Fig. 2c AKO appears highly similar to Fig. 5a AKO-AAV (Zsgreen);
- Fig. 2k and Fig. 3m MMP-2 blots appear to highly similar (also in the associated Source data);
- Fig. 4d WT and KO-AAV(Nrg4) appear to overlap with Extended Data Fig. 4D BATT(WT) on different sides;
- Fig. 5e AKO-AAV(Nrg4) and DKO-AAV(Zsgreen) images all appear highly similar to Extended Data Fig. 4L DKO-BATT(KO) and DKO-BATT(WT), respectively;
- Extended Data Fig. 3M and O appear to use the same Sham and BATT(WT) data, while BATT(KO) values are different by 95 (also in the associated Source data).

The authors have stated that some of the images were incorrectly included in the figures due to errors in image selection, and Extended Data Fig. 3M was not intended for publication.

Due to the high number of errors, the Editors no longer have confidence in the presented data.

Lingfeng Shi, Xiaoli Xu, Yangyang Cheng, Lin Xiang, Junxia Zhang, Lingwei Xiang and Guangda Xiang disagree with this retraction. Yixiang Li, Biying Meng, Jinling Xu, Jiajia Zhang, Kaiyue He and Jiayue Tong have not responded to any correspondence from the publisher about this retraction.

